# Impact of weekly iron-folic acid supplementation on nutritional status and parasitic reinfection among school-age children and adolescents in Sub-Saharan Africa: a systematic review and meta-analysis

**DOI:** 10.3389/fped.2024.1366540

**Published:** 2024-06-17

**Authors:** Shemsu Kedir, Kalkidan Hassen Abate, Bekri Mohammed, Jemal Abafita, Beyene Wondafrash Ademe

**Affiliations:** ^1^ Department of Nutrition and Dietetics, Institute of Health, Jimma University, Jimma, Ethiopia; ^2^ Department of Public Health, College of Medicine and Health Science, Werabe University, Werabe, Ethiopia; ^3^ Department of Nutrition and Dietetics, College of Medicine and Health Science, University of Gondar, Gondar, Ethiopia; ^4^ Department of Economics, Jimma University, Jimma, Ethiopia

**Keywords:** WIFAS, malaria, nutritional status, helminthic reinfection, adolescent

## Abstract

**Background:**

Two significant etiological factors contributing to iron deficiency anemia, and undernutrition posing substantial public health challenges in Sub-Saharan Africa, are soil-transmitted helminths and malaria. This study carried out the effect of weekly iron-folic acid supplementation (WIFAS) on the nutrition and general health of school-age children and adolescents in Sub-Saharan Africa, a systematic review and meta-analysis have been conducted.

**Methods:**

To find pertinent publications for this study, a thorough search was carried out on May 20, 2023, across five databases: Pubmed (MEDLINE), Web of Science, Scopus, Cochrane Library, and Google Scholar. In addition, a search was conducted on August 23, 2023, to capture any new records. The inclusion criteria for the studies were based on school-age children and adolescent populations, randomized controlled trials, and investigations into the effects of WIFAS. The outcomes of interest were measured through anthropometric changes, malaria, and helminthic reinfection**.**

**Results:**

A systematic review of 11 articles revealed that WIFAS significantly decreased the risk of schistosomiasis reinfection by 21% among adolescents (risk ratio = 0.79, 95%CI: 0.66, 0.97; heterogeneity *I*^2^ = 0.00%, *P* = 0.02). However, no significant impact was observed on the risk of malaria reinfection (risk ratio = 1.02, 95%CI: 0.92, 1.13; heterogeneity *I*^2^ = 0.00%, *P* = 0.67) or A. Lumbricoides reinfection (risk ratio = 0.95, 95%CI: 0.75, 1.19; heterogeneity *I*^2^ = 0.00%, *P* = 0.65). Moreover, the analysis demonstrated that there is no significant effect of iron-folic acid supplementation in measured height and height for age *Z*-score (HAZ) of the school-age children (Hedge's g −0.05, 95%CI: −0.3, 0.2; test for heterogeneity *I*^2^ = 0.00%, *P* = 0.7) and (Hedge's g 0.12, 95%CI: −0.13, 0.37; test for heterogeneity *I*^2^ = 0.00%, *P* = 0.36) respectively.

**Conclusion:**

The effectiveness of WIFAS in reducing the risk of schistosomiasis reinfection in adolescents has been demonstrated to be greater than that of a placebo or no intervention. Additionally, the narrative synthesis of iron-folic acid supplementation has emerged as a potential public health intervention for promoting weight change. However, there was no significant association between WIFAS and Ascariasis, trichuriasis, and hookworm. Moreover, the certainty of the evidence for the effects of WIFAS on height and malaria is low and therefore inconclusive. Whereas, the certainty of the evidence for the effectiveness of WIFAS on Schistosomiasis is moderate. Even though the mechanisms need further research WIFAS may be implemented as part of a comprehensive public health strategy to address schistosomiasis in adolescents.

**Systematic Review Registration:**

https://www.crd.york.ac.uk/prospero/display_record.php?ID=CRD42023397898, PROSPERO (CRD42023397898).

## Introduction

Anemia can result from various factors, with iron deficiency accounting for about half of all cases. Hemoglobin disorders, deficiency of Glucose-6-Phospahte-Deficiency (G6PD), problems from delivery, parasite infections such as malaria or helminth infections, and deficits in other critical nutrients are major causes of anemia (1).

In Sub-Saharan Africa, malaria significantly contributes to both illness and death in children prone to anemia through mechanisms such as the direct rupture of infected red blood cells, the immune system's elimination of both infected and uninfected red blood cells, and transient impairment of the bone marrow (1). Infections caused by soil-transmitted helminths (STHs) impact 1.5 billion people, or 24% of the population worldwide. The majority of these infections occur in Sub-Saharan Africa. Treatment and preventive interventions are needed for 108 million youths and more than 654 million teenagers who live in disadvantaged communities with little access to hygiene and sanitation (2).

The World Health Organization (WHO) estimates that nearly two billion people worldwide—roughly 25% of the population—are affected by anemia. About half of all instances of anemia are caused by iron deficiency anemia (IDA), which is responsible for 800,000 deaths globally each year (3). A meta-analysis of 15 studies conducted in 7 countries, recently suggested that providing the nutrients of iron and folic acid to adolescent girls through educational institutions can decrease the prevalence of anemia (4).

The WHO recommends that all pregnant women, reproductive-age women, and children in school (5–12) take intermittent iron supplements when the occurence of anemia is greater than 20% and there are malaria control measures in place (5). However, despite these recommendations, few countries with high anemia prevalence rates have established programs for school-age children, and the region of Africa has little prior experience with these kinds of initiatives.

Furthermore, the impact of supplementing with iron and folic acid on improving nutrition and lowering parasite reinfection is not firmly established. Despite the provision of free IFAS during pregnancy, there is a call for increased interventions targeting adolescents. This systematic review and meta-analysis aim to investigate how the WIFAS impacts school-age children's and adolescents' nutritional status and risk of parasite reinfection in Sub-Saharan Africa.

## Methods

### Searching strategies

The purpose of this meta-analysis and systematic review was to evaluate the effects of WIFAS on nutritional status and parasitic reinfection among children and adolescents. The review encompassed a comprehensive examination of various literature sources, including both published and unpublished research reports, to thoroughly investigate the effects of WIFAS on nutritional status and parasitic reinfection. The systematic search encompassed multiple international databases, such as Web of Science, Scopus, PubMed (MEDLINE), Cochrane Library, and Google Scholar, and the published articles were confined to individuals of school-age children and adolescents in Sub-Saharan Africa.

(((Adolescen*[Title/Abstract] OR “Youth*"[Title/Abstract] OR “primary school"[Title/Abstract] OR “secondary school"[Title/Abstract] OR “teen*"[Title/Abstract] OR “School-age"[Title/Abstract] OR school[Title/Abstract] OR School*[Title/Abstract] OR pediatrics [Title/Abstract] OR pediatric*[Title/Abstract] OR paediatric*[Title/Abstract] OR peadiatric* [Title/Abstract] OR child[Title/Abstract] OR child*[Title/Abstract] OR children*[Title/Abstract] OR Pediatric[Mesh] OR Child[MeSH Terms] OR adolescent[MeSH Terms] OR “Schools"[Mesh]) AND (Iron[Title/Abstract] OR hematinics[Title/Abstract] OR ferrous[Title/Abstract] OR ferric[Title/Abstract] OR hematinic[Title/Abstract] OR haematinic[Title/Abstract] OR haematinics[Title/Abstract] OR “iron compounds"[Title/Abstract] OR “folic acid"[Title/Abstract] OR “Weekly Iron Folic acid supplementation"[Title/Abstract] OR “Dietary supplement*"[Title/Abstract] OR “Iron folic acid supplementation"[Title/Abstract] OR “iron folic acid tablet"[Title/Abstract] OR “Iron-folate supplement*"[Title/Abstract] OR “Iron-folate supplementation"[Title/Abstract] OR “Iron and folic acid supplementation"[Title/Abstract] OR “iron folic acid supplement*"[Title/Abstract] OR “Iron-folic acid"[Title/Abstract] OR Supplementation[Title/Abstract] OR Supplement[Title/Abstract] OR Supplement[Title/Abstract] OR IFAS[Title/Abstract] OR WIFAS[Title/Abstract] OR IFA[Title/Abstract] OR “Iron and Folic-Acid Supplementation"[Title/Abstract] OR Iron[MeSH Terms] OR folic acid[MeSH Terms] OR Dietary supplements[MeSH Terms])) AND (“Randomized controlled trials” OR RCT OR RCTs OR “Clinical Trial” OR “Controlled Clinical Trial” OR “quasi-randomized trials”) AND (Africa, south of the Sahara [MeSH Terms]))). The search terms were combined using Boolean operators “AND'/'OR”. This systematic review included all published studies up to August 23, 2023.

Duplicate articles were removed using the online Rayyan Software, (https://www.rayyan.ai/) after the database search results were combined. This tool was also employed to download the full text of studies for further evaluation.

### Eligibility conditions

The inclusion criteria for this review included studies conducted in Sub-Saharan Africa, studies that have been published in journals with peer reviews were taken into consideration for this review, and only randomized controlled trials (RCTs) and clinical trials were used as study designs. The intervention of interest was iron and/or folic acid supplementation. Language criteria stipulated that articles must be published in English. In this study, the classification of “school-age children” and “adolescents” was based on individuals aged 6–10 years and 10–19 years, respectively. Exclusion criteria encompassed studies related to iron fortification and those lacking specific outcome reporting.

### Outcome measurement

In this study, the main focus was on evaluating the impact of WIFAS on key health indicators, including nutritional status and occurrences of malaria and helminthic reinfection. Measurement of outcomes involved assessing anthropometric measurement through mean and standard deviation calculations. For malaria and helminthic diseases, the prevalence was examined as binary outcomes post-supplementation.

IFAS: this abbreviation indicated that the supplements were received at all times except on once weekly basis.

WIFAS: indicated that the supplements were received only on once weekly basis.

### Data abstraction

A uniform worksheet was employed by two writers (SK and BM) to conduct their independent information collection work. The format for data extraction encompassed details such as the principal author, the year of release, the geographical area where the study was conducted, sample size, frequency of supplementation, age, sex, dose of supplements, outcome measurement, duration of the intervention, and information related to the randomized controlled trials (interquartile range percentage, mean, standard deviation, and median).

### Quality assessment

We used the Joanna Briggs Institute's (JBI) Qualitative Assessment instruments, which are intended for usage in meticulous appraisals of RCTs, to evaluate the methodological integrity of the included research (6). This tool consists of thirteen questions that address the aspects of performance, detection, selection, and attrition bias. Two independent reviewers (SK and BW) meticulously assessed each paper, engaging in discussions to resolve any discrepancies. In cases where disagreements persisted, a third reviewer (KH) was consulted to arbitrate and ensure consistency between the two independent reviewers. We have also contacted authors through email to get some outcome measurements that are mentioned by mean and median, as well as full texts. Each question in the Joanna Briggs Institute (JBI) Critical Appraisal tools was assigned a score: “Yes” received a score of 2, “No” was scored as 0, “Unclear” was denoted as 1, and “Not applicable” was recorded as NA. The cumulative score was used to assess the whole quality of the studies, with scores of 20 and higher being classified as high quality, scores of 13–19 as good quality, and scores of 13 or lower as poorer quality. The detailed results, including the breakdown of scores for each study, can be found in Table 1. Notably, more than half of the studies (63.6%) achieved a high-quality score, while 18% were categorized as lower quality (Table 1).

**Table 1:** In this systematic review and meta-analysis the quality of the included RCTs was evaluated using the JBI quality assessment tool.

S/no	Studies	Was the randomization process indeed random?	Was the assignment to treatment groups hidden?	At baseline, were treatment groups comparable?	Were participants unaware of their assigned treatment?	Did those administering the treatment unaware of the assigned course of action?	Did end-term evaluators blind to the treatment assignment?	Apart from the intervention, are the groups the same?	Was follow-up differences b/n groups?	Was the Intention To Treat (ITT) considered?	Did intervention groups’ results follow the same metrics?	Were results measured with accuracy?	Was the right analysis of statistics applied?	Were the experimental designs suitable	Total Yes
1.	Beasly et.al (Tanzania) (7)	2	0	2	0	0	1	0	2	0	2	1	0	2	10
2.	Olsen et.al (Kenya) (8)	2	2	2	2	2	2	2	2	2	2	1	2	2	24
3.	Lawless et al. (Kenya) (9)	2	2	2	2	2	1	2	2	2	2	1	2	2	22
4.	Mwanakasale et al. (Zambia) (10)	1	1	2	1	1	1	1	1	0	2	1	2	2	8
5.	Nchito et al. (Zambia) (11)	2	2	2	2	2	2	2	2	1	2	1	2	2	22
6.	Baumgartner et al. (South Africa) (12)	2	2	2	2	2	2	2	2	2	2	2	2	2	26
7.	Baumgartner et al. (South Africa) (13)	2	2	2	2	2	2	2	2	2	2	2	2	2	26
8.	Ayoya et al. (Mali) (14)	2	0	2	0	0	1	2	1	0	2	2	2	2	14
9.	Nchito et al. (Zambia) (15)	2	2	2	2	2	2	2	2	1	2	1	2	2	22
10.	Ayoya et al. (Mali) (16)	2	0	2	0	0	1	2	1	0	2	2	2	2	14
11.	Sabine et.al. (Burkina Faso) (17)	2	2	2	0	2	2	2	2	2	2	2	2	2	24

### Statistical analysis

Using an Excel spreadsheet, the retrieved data were input into the computer and transferred into STATA-17 for further analysis. Using the Higgins-I^2^ and Cochran Q statistic, heterogeneity across the data provided was evaluated for twenty-five percent, fifty percent, and seventy percent, respectively, as minimal, moderate, and significant heterogeneity with *p*-values less than 0.05 (18). Utilizing the meta-analysis methodology, the combined impact of WIFAS on nutritional status and parasite reinfection was calculated. To visually represent the subjective presence of variety, a forest plot was also utilized. Sensitivity analysis and sub-group analyses were used to investigate potential discrepancies between trials. The baseline data was compiled using descriptive statistics, such as mean, SD, median, IQR, 95%CI, and proportions. A forest plot with the appropriate hedges, risk ratios, and 95% confidence intervals was used to display the results. Egger's tests were used to evaluate the evidence of publication bias, with a *p*-value of less than 0.05 serving as the threshold for identifying its presence (19, 20). Every outcome's pooled hedges and risk ratios with a 95% confidence interval were applied.

### Registration and reporting

This study was registered with PROSPERO ID: CRD42023397898. Throughout the systematic review procedure, the Preferred Reporting Items for Systematic Reviews and Meta-Analyses (PRISMA) criteria were adhered to (21).

## Result

**Figure 1:**
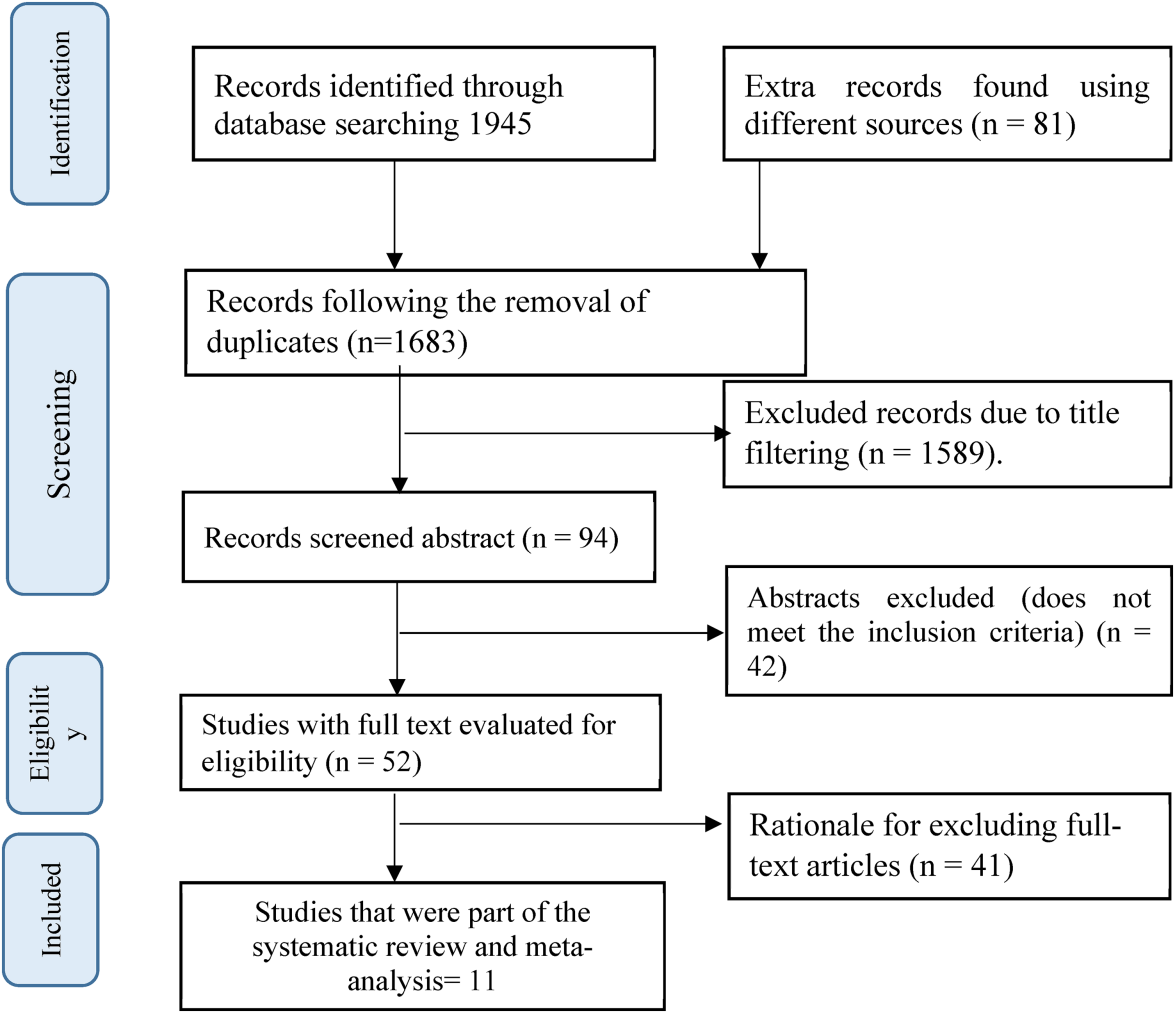
A PRISMA flow diagram was employed to select studies focusing on the impact of IFAS on nutritional outcomes and parasite reinfection.

We identified a total of 2,026 articles (1,945 from PubMed, Web of Science, Scopus, Cochrane Library, and 81 from Google Scholar) through an extensive search across five search engines, enhanced through an extensive inquiry into grey literature, prior evaluations, and the reference list from important works. After excluding 343 duplicates, a review of titles and abstracts against the review objectives and inclusion criteria led to the exclusion of 1,631 articles as irrelevant. After that, the entire texts of the 52 remaining studies were evaluated, and 11 of them satisfied the requirements to be included in the current systematic review and meta-analysis (Figure 1).

### Study characteristics

The current comprehensive reviews were carried out in Sub-Saharan Africa (SSA). Among the included studies, three were conducted in Eastern Africa (7–9), with two in Kenya, and one in Tanzania. Additionally, three studies were conducted in Western Africa (14, 16, 17), with two in Mali, and one in Burkina Faso. Moreover, five studies were carried out in Southern Africa (10–13, 15), with three in Zambia and two in South Africa.

Besides, concerning the frequency of supplementation, four studies were included in weekly and adolescent girls (7, 10, 17, 22), two studies with twice weekly (8, 23), one study with four times per week (12), two studies with five times per week (14, 16), and three studies with daily (9, 11, 15) supplementation.

Regarding the supplement composition, six studies were conducted on iron supplements in the form of ferrous sulfate or ferrous dextran which contains ranging from 50 mg to 65 mg elemental iron and/or folic acid in amounts ranging from 250 μg to 2800 μg (7–10, 17, 23). Furthermore, three studies used a factorial randomized control trial design (11, 12, 15). Among these, two studies employed ferrous dextran with 60 mg elemental iron, 200% of the Recommended Dietary Allowance (RDA) for multivitamins, and an identical placebo (11, 15). Additionally, one study used ferrous sulfate (60 mg elemental iron), 420 mg DHA/80 mg EPA, and a placebo (12). Another study involved ferrous sulfate (120 mg elemental iron) with vitamin A (8.3 mg retinol), a placebo of iron with vitamin A, a placebo of vitamin A with iron, and a double placebo (22). One study focused on Praziquantel (40 mg/kg) alone, Praziquantel with iron (60 mg elemental iron), Praziquantel with iron and multiple micronutrients, and Praziquantel with multiple micronutrients (14). The supplementation period varied within the range of 3 months to 18 months. Only one study was conducted in the form of folic acid (2.8 mg) supplemented with iron (17) (Table 2).

**Table 2:** An overview of the papers that were part of the meta-analysis and systematic review conducted among school-age children in Sub-Saharan Africa, 2023.

s/n	Author, (year, country), reference	Setting and study design	Sex and age	Sample size for intervention group (IG) and control group (CG)	Iron dose	Folic acid dose	Frequency and duration of supplement	Outcome measurement
1	Beasly, et.al. (Tanzania) (7)	School; RCT	Female, 12–18 years	IG = 50 CG = 57	400 mg FS	–	Weekly for 4 month	Diarrhea, Malaria, and Wt. change
2	Olsen, et.al. (Kenya) (8)	Community RCT	4–15 years	IG = 108 CG = 92	60 mg EI	–	Twice weekly for 12 month	IP
3	Lawless et al. (Kenya) (9)	School; RCT	6–11 years	IG = 44 CG = 42	150 mg EI	–	Daily for 3.2 month	Wt. & Ht change, HAZ, appetite score, and Cough
4	Mwanakasale et al. (Zambia) (10)	School; RCT	Male, 9–15 years	IG = 80 CG = 87	200 mg FS	–	Weekly for 9 month	IP
4	Mwanakasale et al. (Zambia) (10)	School; RCT	Female, 9–15 years	IG = 73 CG = 84	200 mg FS	–	Weekly for 9 month	IP
5	Nchito et al. (Zambia) (11)	School; RCT	7–15 years	IG = 98 CG = 42	60 mg EI	–	Daily for 10 month	IP
6	Baumgartner et al. (South Africa) (12)	School; FRCT	6–11 years	IG = 80 CG = 80	50 mg EI	–	4*/week for 8.5 month	Wt. & Ht change, BAZ, and HAZ
7	Leenstra et.al. (Kenya) (22)	School; RCT	Female, 12–18 years	IG = 80 CG = 109	120 mg EI	–	Weekly for 5 month	Malaria
8	Olsen et.al. (Kenya) (23)	Community RCT	4–15 years	IG = 108 CG = 92	60 mg EI		Twice weekly for 12 months	IP
9	Ayoya et al. (Mali) (14)	School; RCT	7–12 years	IG = 309 (3 groups) CG = 97	60 mg EI	–	5 days/week for 3 months	Malaria
10	Nchito et al. (Zambia) (15)	School; RCT	7–15 years	IG = 101 CG = 101	60 mg EI	–	daily for 10 month	IP
11	Sabine et.al. (Burkina Faso) (17)	Community	Female, 10–19 years		60 mg EI	2.8 mg	Weekly for 18 months	Malaria

RCT, randomized control trial; FRCT, factorial randomized control trial; IG, intervention group; CG, control group; EI, elemental iron; FS, ferrous sulphate; Wt, weight; Ht, height; IP, intestinal parasitosis; BAZ, body mass index for Z score; HAZ, height for age Z score; SAC, school age children.

## Effect of IFAS on anthropometric measurement-narrative synthesis

### Appetite score

In a study on the effects of iron supplementation on anemic Kenyan primary school students, Lawless JW (9) found that giving the kids iron supplements significantly increased their appetite. This improvement was observed both in terms of increased energy intake during snacks and children's self-reported appetite when compared to those receiving a placebo. 10% of the daily estimated energy intake for children in the same age group living in other parts of Kenya was derived from the increased energy intake from the snacks.

### Weight change

In this review, three studies were included, of which two studies (Beasley NR, et.al, 2000 and Lawless JW, et.al, 1994) (7, 9) measured the difference of weight by using mean and standard deviation. The findings of these studies indicated that iron supplementation significantly improved the mean weight and gain greater weight compared to the placebo, with values of 2.4 kg ± 2.3 vs. 1.6 kg ± 0.8 and 1.6 kg ± 0.5 vs. 0.7 kg ± 0.4, respectively. Meanwhile, Jeannine B. et al., measured the change in weight using the median, and the findings showed that iron supplementation significantly increased the median weight compared to the placebo (12).

### Height change

Lawless JW, et.al. and Jeannine B. et al. (9, 12) studies measured the difference in height by using mean and standard deviation and the findings of these studies indicated that iron supplementation significantly improved the mean height compared to the placebo.

### HAZ change

Lawless JW, et.al. and Jeannine B, et.al. studies (9, 12) measured the difference in height-for-age *Z*-score using mean and standard deviation. The results of these studies showed that children who took iron supplements changed their mean height for age in a significantly different way (*P* < 0.05) than the control group. The kids' mean height for age declined overall in both groups, indicating that they were not keeping up with the NCHS height growth curve and were generally lagging in their linear growth.

### BAZ change

In 2012, Jeannine B. and colleagues (12) evaluated the alteration in body mass index for age *Z*-score by analyzing the mean and standard deviation. The results indicated no significant difference between the group receiving iron supplementation and the placebo group (12).

### Effect of IFAS on anthropometric measurement-meta analysis

#### Height change

**Figure 2:**
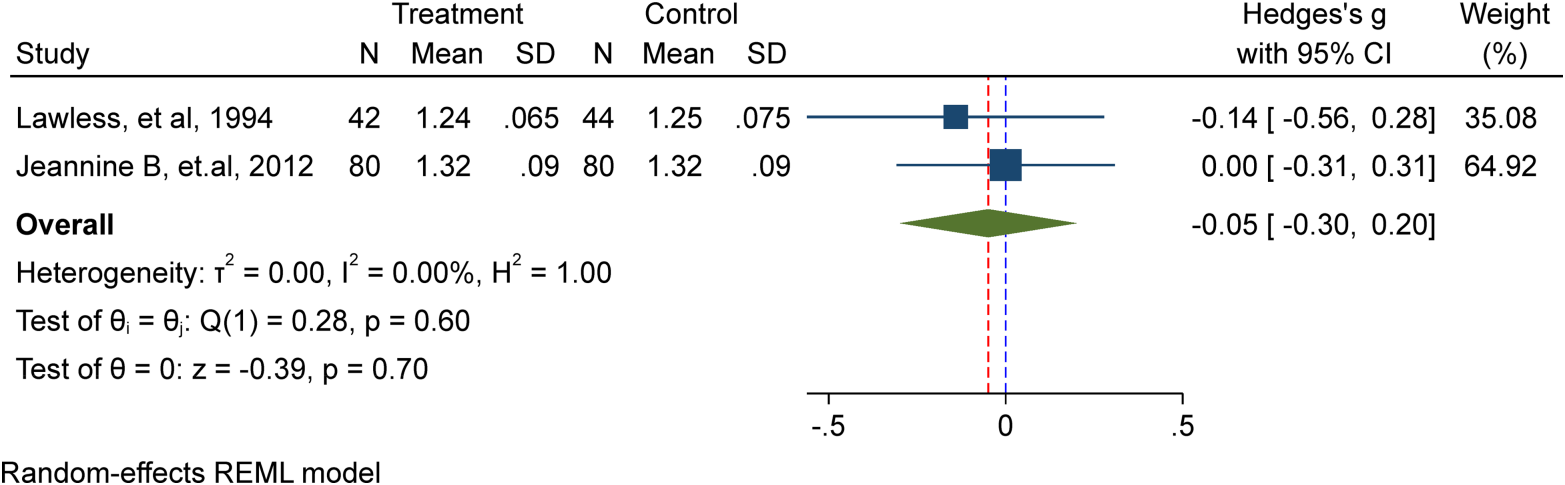
Meta-analysis of the effect of IFAS on height.

In this meta-analysis, two studies (9, 12) comprising 246 school-age children, including adolescents, were included. Among them, 122 received iron supplementation, while 124 were part of the placebo/non-intervention group. The results of the investigation showed that iron supplementation has no discernible impact on school-age children's height (Hedge's g −0.05, 95%CI: −0.3, 0.2; test for heterogeneity *I*^2^ = 0.00%, *P* = 0.7) (Figure 2).

#### Height for Age Z-score (HAZ)

**Figure 3:**
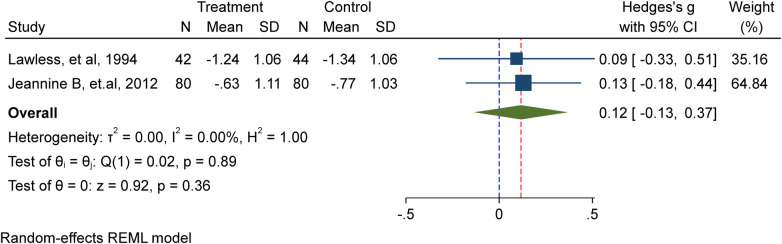
Meta-analysis of the effect of IFAS on height for age Z score (HAZ).

In this meta-analysis, a total of two studies (9, 12) involving 246 school-age children, including adolescents, were included. Among them, 122 received iron supplementation, while 124 were part of the placebo/non-intervention group. The investigation showed that the height for age *Z*-score (HAZ) of school-age children is unaffected by iron supplementation in a substantial way (Hedge's g 0.12, 95%CI: −0.13, 0.37; test for heterogeneity *I*^2^ = 0.00%, *P* = 0.36) (Figure 3).

### Effect of IFAS on common morbidity reinfection-narrative synthesis

#### Malaria

Five studies assessed the impact of IFAS on malaria reinfection, and among these four studies (7, 9, 17, 22) reported that there was no significant association between children who received IFAS and their counterparts. However, according to Mohammed AG et al., the study identified sixteen individuals in the P + Fe and P + Fe + MM groups that received iron, compared with nine individuals in the P and P + MM groups that were not provided with additional iron (14). When comparing the iron group to the non-iron group, the relative risk (RR) was 1.66. There were only 3 children with high-density parasitemia (≥5,000 parasites/ml), and all of those cases happened after 6 weeks. Among children with malaria, 86% exhibited serum ferritin concentrations within the normal range (≥12 mg/L), suggesting a potential association between iron saturation and increased malaria incidence.

### Effect of WIFAS on malaria-meta analysis

**Figure 4:**
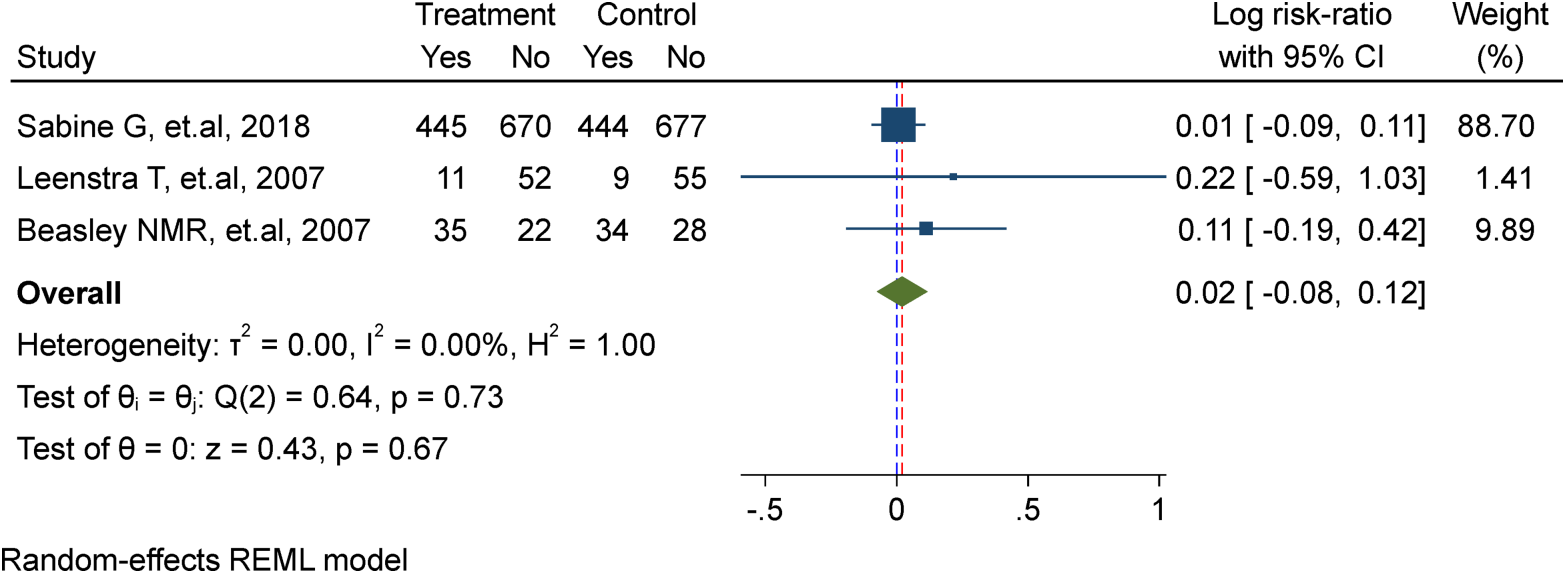
Meta-analysis of the effect of once-weekly IFAS on malaria.

In this meta-analysis, three studies were included (7, 17, 22), comprising a total of 2,482 adolescent girls. Among them, 1,233 received weekly iron supplementation in the treatment group, while 1,243 were assigned to the placebo/non-intervention group. The analysis demonstrated that there is no substantial effect of weekly iron supplementation in lowering the risk of re-infection of malaria (Risk ratio = 1.02, 95%CI: 0.92, 1.13; test for heterogeneity *I*^2^ = 0.00%, *P* = 0.67) (Figure 4).

### Effect of IFAS on helminthic reinfection-narrative synthesis

#### Schistosomiasis

At 6 months (*P* < 0.001) and 9 months (*P* < 0.001) of supplementation, boys in the treatment group had considerably less S. haematobium reinfection than boys in the control group, according to research by Victor M. et al. (10). Furthermore, only six months after supplementation, girls in the intervention group showed significantly lower S. haematobium reinfection intensity than girls in the control group (*P* = 0.018). At the 6-month follow-up, boys in the intervention group had a 42% lower chance of contracting S. haematobium again than those in the control group (ARR = 0.58, 95%CI: 0.39, 0.86). Nevertheless, Olsen et al. (23) found no evidence of a significant correlation between the impact of IFAs and reinfection with Schistosomiasis (23).

### Ascaris Lumbricoides

Four trials were examined in this study to see how IFAS affected Ascaris Lumbricoides reinfection. Out of these, three studies (8, 11, 23) did not show a significant association between IFAS and A.Lumbricoides reinfection. However, compared to non-geophagous children, geophagous children exhibited a significantly greater prevalence of A. Lumbricoides infection (53.7% vs. 30.6%, *P* = 0.024) among those with hemoglobin levels <130 g/L, according to one study (Nchito et al.) (15). Therefore, iron supplementation dramatically lowers the incidence of geophagy (*P* = 0.044).

### Hookworm and trichuris trichiura

Olsen A. et al.'s study showed that iron supplementation did not affect children's rates of reinfection or the severity of Hookworm and Trichuris trichiura infections (23).

### Effect of WIFAS on schistosomiasis-meta analysis

**Figure 5:**
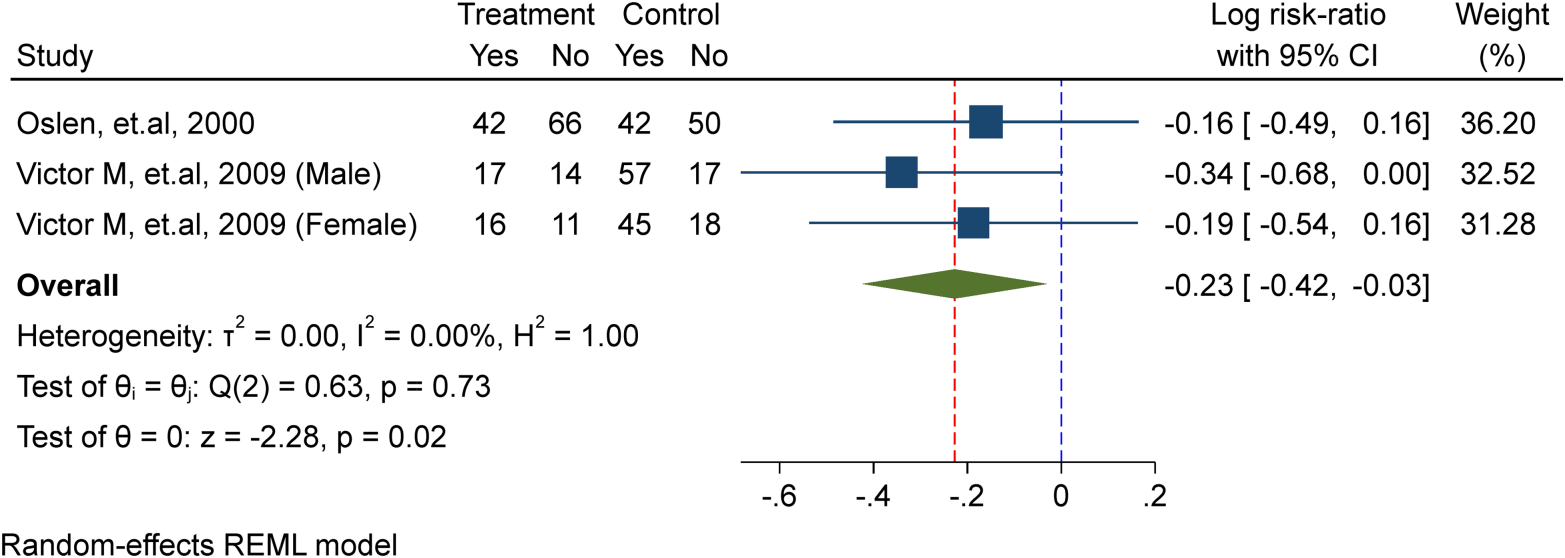
Meta-analysis of the effect of once-weekly IFAS on schistosomiasis.

In this meta-analysis, two studies (10, 23) were included, comprising a total of 395 school-age children, including adolescent girls. Of these, 229 were allocated to the placebo/non-intervention group and 166 were part of the treatment/intervention group, which received weekly iron supplements. The analysis showed that weekly iron supplementation significantly lowers the probability of schistosomiasis re-infection by 21% (probability ratio = 0.79, 95% confidence interval).CI: 0.66, 0.97; *I*^2^ = 0.00%, *P* = 0.02) in the test for heterogeneity (Figure 5).

### Effect of IFAS on ascariasis-meta analysis

**Figure 6:**
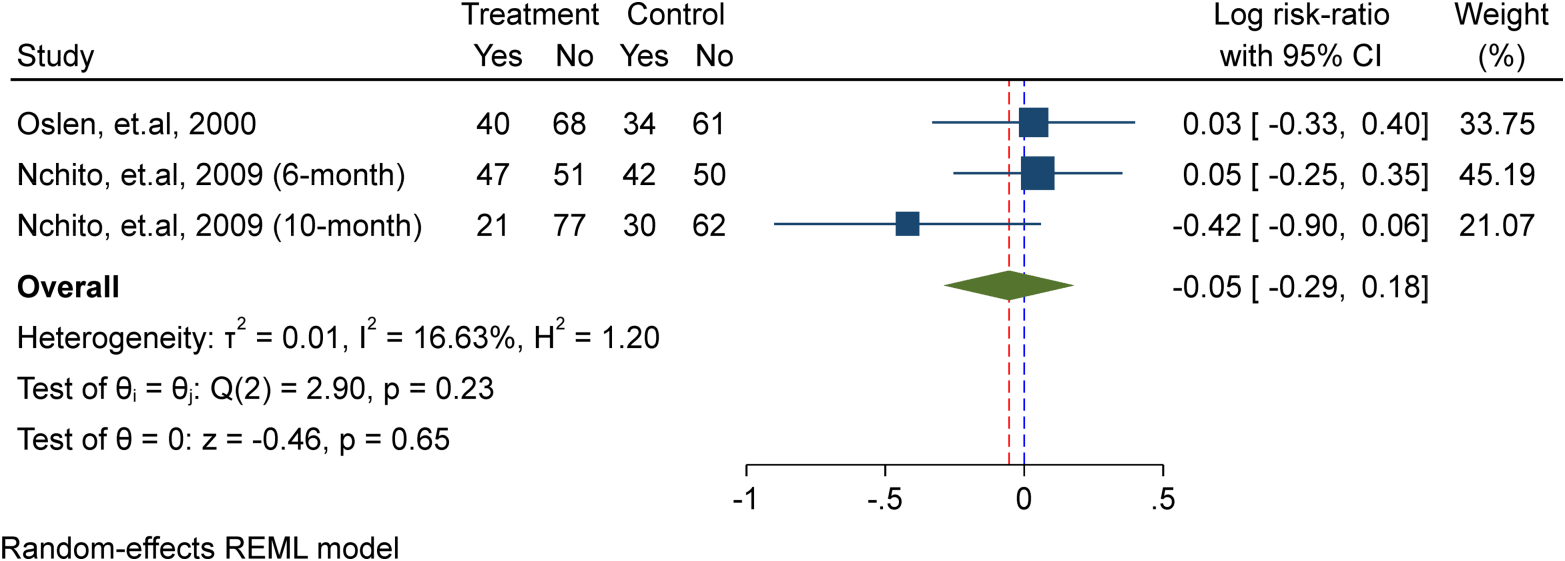
Meta-analysis of the effect of IFAS on Ascaris Lumbricoides.

In this meta-analysis, two studies were included (11, 23), comprising a total of 583 school-age children, including adolescent girls. Among them, 304 received iron supplementation in the treatment group, while 279 were assigned to the placebo/non-intervention group. The results of the analysis showed that iron supplementation had no discernible effect on the risk of A. lumbricoid re-infection (Risk ratio = 0.95, 95%).CI: 0.75, 1.19; *I*^2^ = 0.00%, *P* = 0.65 for the test of heterogeneity) (Figure 6).

### Certainty of evidence

We took into account variables including publication bias, substantial impact, dose-effect relationships, and confounding in addition to the possibility of bias, inconsistencies, indirectness, inaccuracy, and uniformity. The risk of bias was assessed using the Cochrane risk of bias tool for 2019, which included criteria such as intention-to-treat analysis, blinding/masking of endpoint examiners, blinding/masking of the intervention, sequence creation, and independence from other pitfalls (24). The variability (*I*^2^) of the whole impact in the meta-analysis was used to investigate inconsistencies. The external validity, applicability, and any departures from the study topic were carefully examined about indirectness. Wide confidence intervals, such as those that show a null effect and greater relative risk (RR > 0.75 or >1.25), were used to examine imprecision. We also evaluated publishing biases. Our results lead us to advocate, in a modest way, weekly iron-folic acid supplementation (WIFAS), which lowers schistosomiasis and anemia while raising serum ferritin and hemoglobin levels (Table 3).

**Table 3:** The evaluation of the selected articles’ evidence of degree of certainty during the meta-analysis.

Outcomes	Number of studies	Number of study participants	Study design	Risk of bias (RoB)	Inconsistency (heterogeneity)	Indirectness	Imprecision	Publication bias (EGGERS test with *P*-values)	Other considerations (large effect, dose-response, or confounding	Effect size [hedges (H) or RR]	Certainty of evidence
Height	2	246	RCT	Low	Low	No	Concern	No	Concern	H = −0.05 (−0.3, 0.2)	Low
HAZ	2	246	RCT	Low	Low	No	Concern	No	No	H = 0.12 (−0.13, 0.37)	Moderate
Malaria	5	2,482	RCT	Serious	Low	No	Concern	No	No	RR = 1.02 (0.92, 1.13)	Low
A.Lumricoides	4	583	RCT	Low	Low	No	Concern	No	No	RR = 0.95 (0.75, 1.19)	Moderate
Schistosomiasis (once-weekly)	2	395	RCT	Low	Low	Low	Concern	No	No	RR = 0.79 (0.66, 0.97)	Moderate

## Discussion

The current study incorporated 11 randomized trials in this systematic review to examine the impact of weekly iron-folic acid supplements (WIFAS) on numerous health indicators including anthropometric change, helminthic infection, and malaria. The trials were distributed across Southern Africa (five studies), East Africa (three studies), and West Africa (three studies).

Our systematic review of two studies regarding weight change indicated that iron supplementation significantly improved the mean weight and gained greater weight compared to the placebo. However, the pooled analysis of height and HAZ score had no significant association with IFAS. Similarly, a review of studies found no significant link between iron supplementation and measures like WAZ, HAZ, and circumferences of the arm. Some studies showed increased weight in malaria-prone regions and lower growth in developed countries where supplements were given for over six months (25).

The study found that WIFAS significantly reduced schistosomiasis reinfection. This aligns with a previous study by Morales-Suarez-Varela et al. (2019) which showed that micronutrient supplementation decreased infestation with Schistosoma spp by 1.33 times compared to a placebo. The supplementation was also more effective in reducing infestation with Schistosoma mansoni (1.3 times) and Schistosoma haematobium (1.62 times) compared to the placebo. These findings show a direct correlation between supplements and a decrease in infestation. When children and adolescents receive micronutrient supplements, the prevalence of Schistosoma spp. is reduced (26).

The present study revealed that there was no significant association between WIFAS/IFAS and Ascariasis, trichuriasis, and hookworm. According to WHO peer-reviewed publications, WIFAS and regular deworming help to prevent iron and folate deficiencies. In this report anemia prevalence fell from 38% to 18%, iron deficiency fell from 23% to 8% and the prevalence of iron deficiency anemia was reduced to 4%. The level of moderate or heavy infestation of any soil-transmitted worm infection was reduced to less than 1% (27). This idea indicated that iron supplements alone may not reduce STH/NTD.

The current study found no significant impact of IFAS on the risk of malaria reinfection. Previous research by Clark et al. suggested that Iron supplementation may heighten vulnerability to malaria by stimulating the development of immature red blood cells, which P. falciparum favors. The study by Goheen et al. also showed significant changes in RBCs after iron supplementation, supporting this hypothesis (28, 29).

However, the World Health Organization (WHO) and Neuberger et al. recommend that only in regions where malaria is highly prevalent, in addition to efficient malaria management and preventive measures, should iron supplements be used. This is because conducting iron supplementation trials without concurrent antimalarial interventions is difficult, as these interventions could potentially obscure results (30, 31). However, there is still a lack of a thorough understanding of the cellular interactions among irons and malaria vulnerability, and it is difficult to undertake supplementation with iron trials missing simultaneous antimalarial therapies. This difficulty arises because such interventions could potentially obscure results, complicating the interpretation of findings.

Recent evidence, however, indicates that iron supplementation, when paired with routine malaria prevention and tracking, may be safe for kids. It is advised to use intermittent regimens, which include giving iron supplements a single time, twice, or three times a week on non-consecutive days, as a practical way to increase children's iron consumption with fewer negative consequences (1).

## Limitations of the study

Our study is subject to inherent limitations related to the impacts of intermittent IFAS on anthropometric changes, malaria reinfection, and helminthic reinfection which broadens the scope of the study. The analysis faces challenges owing to the use of various tools to assess specific domains, complicating comparisons between intervention outcomes. Complexity is increased by significant variations in the intervention's design, which affect the dosage, kind, and duration of iron supplements. Establishing ideal dosage, degree, or time for improved or reduced outcomes for schistosomiasis, and malaria remains elusive. The duration of the intervention for detecting changes in anthropometric outcomes requires consideration. Additionally, the potential influence of other micronutrients remains unclear in some studies. Nine research were deemed to have a low risk of bias, whereas two studies had a high risk of bias, the researchers acknowledge the possibility of missing relevant studies.

## Conclusion

The present study has shown that weekly iron-folic acid supplementation is more effective than either placebo or no intervention at lowering the likelihood of schistosomiasis reinfection in adolescents. Additionally, the narrative synthesis of iron supplementation has surfaced as a viable public health strategy to encourage weight increase. However, there was no significant association between WIFAS and Ascariasis, trichuriasis, and hookworm. Moreover, the certainty of the evidence for the effects of iron-folic acid supplementation (IFAS) on height and malaria is low and therefore inconclusive. Whereas, the certainty of evidence for the effectiveness of WIFAS on Schistosomiasis is moderate.

Therefore, WIFAS may be implemented as part of a comprehensive public health strategy to address schistosomiasis. However, more research is needed to understand the mechanism and determine the best way to provide iron supplementation alone to people with NTDs in order to maximize the benefits and minimize the risks in adolescents.

## Data Availability

The original contributions presented in the study are included in the article/Supplementary Material, further inquiries can be directed to the corresponding author.
